# Supplementary material for: Discovery of New Anti-Cancer Agents against Patient-Derived Sorafenib-Resistant Papillary Thyroid Cancer
Source: Int J Mol Sci. 2023 Nov 16;24(22):16413. doi: 10.3390/ijms242216413 (PMC10671409; doi:10.3390/ijms242216413)
Supplement: Supplementary file 1 [file ijms-24-16413-s001.zip › ijms-2648229-SI.pdf]

Supplementary Table

Supplementary Table S1. Primer sequences for qRT-PCR.

Gene	Forward primer (5'-3')	Reverse primer (5'-3')
SERCA1	GTGATCCGCCAGCTAATG	CGAATGTCAGGTCCGTCT
SERCA2	GGTGGTTCATTGCTGCTGAC	TTTCGGACAAGCTGTTGAGG
SERCA3	GATGGAGTGAACGACGCA	CCAGGTATCGGAAGAAGAG
α-tubulin	CGGGCAGTGTTTGTAGACTTGG	CTCCTTGCCAATGGTGTAGTGC

Supplementary Methods

S1. Study design and Ethical considerations

This study was a retrospective, which solitary centre analysis of patients with PTC, detail information was described in our previous study [1–3] and supplementary methods. All procedures involving patients were performed in accordance with the institutional ethical standards, all applicable local/national regulations, and guidelines of the 1964 Helsinki Declaration and its later amendments. In accordance with the Bioethics and Safety Act of Korea, formal written consent was not required for this type of retrospective, observational analysis. The study protocol was approved by the Institutional Review Board (IRB) of Severance Hospital, Yonsei University College of Medicine (IRB protocol: 3-2022-0331). Cell samples were isolated from patient specimen at the Severance Hospital, Yonsei University College of Medicine, Seoul, Korea.

S2. Patients

S2.1. Patient 1

YUMC-S-P1 was 53-year-old man with papillary thyroid cancer. This patient had bilateral thyroid tumors with extrathyroidal extension. This patient underwent bilateral total thyroidectomy and bilateral modified radical neck dissection with central compartment neck dissection. Surgical findings showed that the tumor invaded the recurrent laryngeal nerve and was removed by careful shaving. After surgery, she was given 3 times high-dose radioiodine ablation therapy. Currently, radiologic examination and thyroid hormone tests are being followed without recurrence.

S2.2. Patient 2 and 3

YUMC-R-P5 and -P6 were 52 and 57-year-old woman and man with papillary thyroid cancer. This patient had multiple tumors and extensive extrathyroidal extension. This patient underwent bilateral total thyroidectomy with central compartment neck dissection. One year after surgery, metastasis to the mediastinum and right lateral cervical lymph nodes was confirmed, and she underwent mediastinal dissection through partial sternotomy and right modified radical neck dissection. The specimens for culture were obtained after the last operation. This patient underwent sorafenib after which the disease progression was confirmed in the sorafenib drug response evaluation. Cancer recurrence and metastasis were caused after sorafenib prescribed.

S3. mRNA-Seq data

We preprocessed the raw reads from the sequencer to remove low quality and adapter sequences before analysis and aligned the processed reads to the *Homo sapiens* genome assembly (GRCh37) using HISAT v2.1.0 [4]. HISAT utilizes two types of indexes for alignment: a global, whole-genome index, and tens of thousands of small local indexes. Both are constructed using the same Burrows–Wheeler transform (BWT) or graph FM index (GFM) as Bowtie2. Because of the use of these efficient data structures and algorithms, HISAT generates spliced alignments several times faster than Bowtie and the widely used BWA. The reference genome sequence of Homo sapiens (GRCh37) and annotation data were downloaded from the National Center for Biotechnology Information (NCBI). Then, transcript assembly of known transcripts was processed using StringTie v2.1.3b [5,6]. Based on these results, expression abundance of transcript and gene were calculated as read count or fragments per kilobase of exon per million fragments mapped (FPKM) value per sample. The expression profiles were used for additional analyses, such as of differentially expressed

genes (DEGs). In groups with different conditions, differentially expressed genes or transcripts were filtered through statistical hypothesis testing.

References

1. Chang, H.S.; Kim, Y.; Lee, S.Y.; Yun, H.J.; Chang, H.J.; Park, K.C. Anti-cancer serca inhibitors targeting sorafenib-resistant human papillary thyroid carcinoma. *Int J Mol Sci* **2023**, *24*.
2. Kim, S.M.; Park, K.; Lim, J.H.; Yun, H.J.; Kim, S.Y.; Choi, K.H.; Kim, C.W.; Lee, J.H.; Weicker, R.; Pan, C.H. *et al.* Potential therapeutic agents against paclitaxel-and sorafenib-resistant papillary thyroid carcinoma. *Int J Mol Sci* **2022**, *23*.
3. Yun, H.J.; Kim, M.; Kim, S.Y.; Fang, S.; Kim, Y.; Chang, H.S.; Chang, H.J.; Park, K.C. Effects of anti-cancer drug sensitivity-related genetic differences on therapeutic approaches in refractory papillary thyroid cancer. *Int J Mol Sci* **2022**, *23*.
4. Kim, N.; Kim, K.H.; Lim, W.J.; Kim, J.; Kim, S.A.; Yoo, H.J. Whole exome sequencing identifies novel de novo variants interacting with six gene networks in autism spectrum disorder. Genes (Basel) **2020**, 12.
5. Pertea, M.; Pertea, G.M.; Antonescu, C.M.; Chang, T.C.; Mendell, J.T.; Salzberg, S.L. Stringtie enables improved reconstruction of a transcriptome from rna-seq reads. Nat Biotechnol **2015**, 33, 290-295.
6. Pertea, M.; Kim, D.; Pertea, G.M.; Leek, J.T.; Salzberg, S.L. Transcript-level expression analysis of rna-seq experiments with hisat, stringtie and ballgown. Nat Protoc **2016**, 11, 1650-1667.
